# Stochastic resonance enhances the rate of evidence accumulation during combined brain stimulation and perceptual decision-making

**DOI:** 10.1371/journal.pcbi.1006301

**Published:** 2018-07-18

**Authors:** Onno van der Groen, Matthew F. Tang, Nicole Wenderoth, Jason B. Mattingley

**Affiliations:** 1 Neural Control of Movement Laboratory, Department of Health Sciences and Technology, ETH Zurich, Zurich, Switzerland; 2 Queensland Brain Institute, The University of Queensland, St Lucia, Queensland, Australia; 3 School of Psychology, The University of Queensland, St Lucia, Queensland, Australia; Duke University, UNITED STATES

## Abstract

Perceptual decision-making relies on the gradual accumulation of noisy sensory evidence. It is often assumed that such decisions are degraded by adding noise to a stimulus, or to the neural systems involved in the decision making process itself. But it has been suggested that adding an optimal amount of noise can, under appropriate conditions, enhance the quality of subthreshold signals in nonlinear systems, a phenomenon known as *stochastic resonance*. Here we asked whether perceptual decisions made by human observers obey these stochastic resonance principles, by adding noise directly to the visual cortex using transcranial random noise stimulation (tRNS) while participants judged the direction of coherent motion in random-dot kinematograms presented at the fovea. We found that adding tRNS bilaterally to visual cortex enhanced decision-making when stimuli were just below perceptual threshold, but not when they were well below or above threshold. We modelled the data under a drift diffusion framework, and showed that bilateral tRNS selectively increased the drift rate parameter, which indexes the rate of evidence accumulation. Our study is the first to provide causal evidence that perceptual decision-making is susceptible to a stochastic resonance effect induced by tRNS, and to show that this effect arises from selective enhancement of the rate of evidence accumulation for sub-threshold sensory events.

## Author summary

Noise is usually thought of as being detrimental for perception and decision-making, but recent work has revealed that under certain circumstances simple detection performance can be enhanced when an optimal amount of noise is applied to the visual cortex non-invasively using tRNS. Here we asked whether this stochastic resonance effect also applies to a higher level perceptual decision-making task. We found that adding an optimal level of neural noise to the visual cortex bilaterally enhanced decision-making, specifically for below-threshold stimuli, consistent with a stochastic resonance effect. Computational modelling under a hierarchical drift diffusion framework revealed that the enhancement of observers’ decision making with optimal noise arose from an increase in the rate of perceptual evidence accumulation. The findings provide new evidence in support of stochastic resonance as a neural mechanism through which weak stimuli can influence perceptual decisions, and suggest a novel target for interventions in neurological patients.

## Introduction

**Fig 1:**
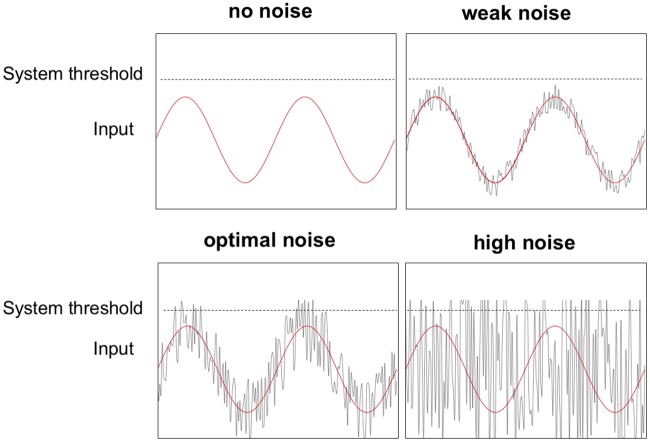
Stochastic resonance occurs when an optimal level of noise is added to a subthreshold signal. In this example the signal alone (red sinusoid) remains below the perceptual threshold (dotted line). Adding an optimal amount of noise (gray line) periodically raises the stimulus above the system threshold. If the added noise is too weak, the threshold is not crossed. Conversely, if the noise is too strong the signal remains buried and cannot be discriminated from the noise [14].

Noise is an intrinsic property of all biological systems [1]. Typically, noise is viewed as being detrimental for neuronal computations and the behaviors they regulate [1, 2], including decision-making [3]. A key limiting factor in decision-making arises from noisy representations of sensory evidence in the brain [4, 5]. On this view, noisy sensory information representations are not optimal, and this leads to errors in decisions. However, small amounts of noise added to a nonlinear system can increase stimulus quality by increasing the signal-to-noise ratio (SNR)[6]. This phenomenon is known as *stochastic resonance* (Fig 1), and its expression has been demonstrated in different sensory modalities [7–9]. Stochastic resonance occurs when an optimal amount of noise is added to a sub-threshold signal, which makes the signal cross a threshold and therefore enhances detection performance (Fig 1) [10–13].

**Fig 2:**
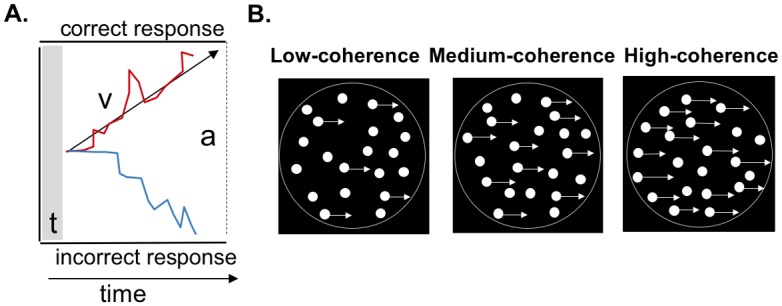
**A**: Schematic of the drift diffusion modelling (DDM) framework used to model perceptual decision-making in the dot motion task. In the model, evidence is accumulated over time until a response boundary is crossed. *t* is the non-decision time, which includes the time taken to execute a motor response. *v* is the drift rate, which reflects the rate at which sensory evidence is accumulated. This parameter is taken as an index of the quality of sensory information. *a* represents the boundary separation (*correct* at the top, *incorrect* at the bottom), indicating how much information is needed to make a decision. **B**: Schematic of the random dot-motion task in which participants judged whether signal dots moved on average to the left or right. Task difficulty was titrated by altering the proportion of coherently moving dots (shown with arrows attached, for purposes of illustration) amongst randomly moving dots. In this example the coherent motion is rightward, but in the experiment the dots were equally likely to move toward the left or right. The circles surrounding the dot stimuli are shown here for illustration only, and were not present in the actual displays.

Neurophysiologically, adding an optimal amount of noise to a subthreshold signal pushes otherwise silent sensory neurons above the spiking threshold [7, 15, 16]. A common way of adding noise in a stochastic resonance context is to add it directly to the sensory stimulus. In such cases, however, the noise might simply increase peripheral receptor sensitivity [17], which would not address the question of whether central neural processes in decision-making are sensitive to a stochastic resonance mechanism. Recently, we showed that it is possible to induce a stochastic resonance effect in a simple detection task when noise is added to the visual cortex with transcranial random noise stimulation (tRNS [18]). Although the underlying neural mechanisms are not completely understood [19], single unit recordings in visual cortex have revealed an increase in the SNR of neuronal spiking when an optimal level of noise is applied to a visual stimulus [20], consistent with a stochastic resonance mechanism. This is likely due to the recruitment of voltage-gated sodium channels by the noise [21–23]. Our previous study [18], together with several related investigations [24, 25], point to stochastic resonance as an underlying mechanism by which non-invasive brain stimulation can enhance behavioural performance when it is applied concurrently during task performance. Here we go beyond these findings by asking whether higher-level perceptual decisions in a random-dot-motion (RDM) task (see Fig 2) are susceptible to a stochastic resonance effect. The RDM task has been widely used in studies of perceptual decision-making, and has well characterized neural correlates [26, 27]. A recent study showed that RDM judgements are affected when noise is added peripherally to a visual display [28], but it remains unclear whether an analogous effect arises for noise administered centrally (i.e., to the cerebral cortex).

In addition to measuring the influence of central noise on perceptual decisions, we also investigated which aspects of the decision process itself are sensitive to stochastic resonance using drift diffusion modelling (DDM; see Fig 2 [29, 30]). Such modelling approaches have been very successful in describing both human and animal behavior [29]. Under the DDM framework, performance improvements can occur via a change in the decision criterion (i.e., the bound separation), or through an increase in the rate of evidence accumulation (i.e., the drift rate; [29]). In the current study, an increase in bound separation would suggest that the stochastic resonance effect is driven by a change in the decision criterion, whereas an increase in drift rate would suggest an improvement in the quality of sensory evidence on which the decision is based. In addition, if the stochastic resonance model applies to perceptual decision-making, then the addition of relatively small amounts of noise should enhance discrimination performance for coherent motion trials in which the signal is just below threshold, but not for trials in which the signal is well below or above threshold [8].

Finally, previous brain imaging studies have shown that visual motion stimuli elicit bilateral activation of extrastriate visual cortex [31–33]. By contrast, application of non-invasive brain stimulation over left hemisphere visual areas has been shown to have larger effects on motion discrimination than equivalent stimulation over right hemisphere regions [34–36]. We therefore applied tRNS over visual cortex bilaterally and unilaterally (left and right), across separate experiments, to determine whether any stochastic resonance effect can be induced by stimulating the two hemispheres in combination or alone.

Our results show that adding an optimal amount of noise to the visual cortex bilaterally enhances perceptual decision making in the RDM task, consistent with the stochastic resonance hypothesis. Performance deteriorated with larger amounts of noise, and the effect was not evident during unilateral hemispheric stimulation. Modelling of observers’ responses under the drift diffusion framework revealed that the improvement in performance with optimal noise was associated with a reliable increase in the drift rate parameter, implying an increase in the rate of evidence accumulation.

## Results

### Experiment 1: Effect of bilateral visual cortex stimulation on perceptual decision-making

**Fig 3:**
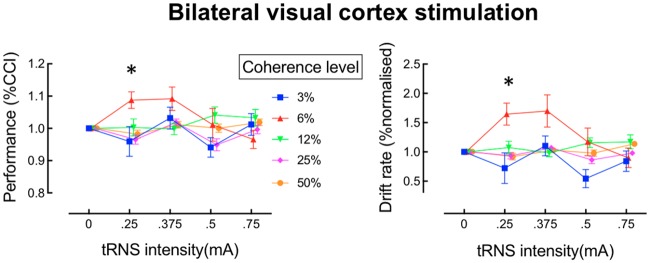
Effects of transcranial random noise stimulation (tRNS) on perceptual decision-making in the dot-motion discrimination task for bilateral stimulation. The left panel shows performance for each motion coherence level as a function of tRNS intensity. The right panel shows the drift rate derived from modelling of the data shown in the corresponding plot to the left. *p_corrected_ < 0.05.

In Experiment 1, we stimulated the visual cortex bilaterally with tRNS in 15 participants. The coherence levels of 3% and 6% were subthreshold for both the group as a whole, and for the individual observers (average detection performance < 63%), i.e., performance was below the detection threshold, which corresponded to 75% correct in our task. For each observer we determined an individual discrimination threshold in the noise-free trials, and showed that this was above 6% coherence in all individuals. As shown in the left panel in Fig 3, for the 6% coherence condition, which was just below threshold in the no-tRNS condition, motion discrimination performance improved when tRNS was applied at a relatively low intensity, whereas performance remained unaffected for the other coherence levels and noise intensities. For the analysis, we calculated the group %correct-choice-index (%CCI) for each coherence level and each tRNS intensity by dividing the %correct motion-direction responses under tRNS by the %correct responses when no tRNS was applied (baseline), as given in the following formula:

(%CCI)=%Corr(i)/%Corr(zeronoise)

where *i* denotes each of the 4 tested noise intensities.

There was a significant interaction between coherence level and tRNS-intensity (F(12,156) = 2.47 p < 0.01, Cohen’s f = 0.43). To isolate the source of this interaction, one-way ANOVAs were conducted for each coherence level separately. For the 6% coherence condition only (red symbols in Fig 3), performance was significantly affected by the different tRNS intensities (F(3,39) = 3.56 p = 0.02 Cohen’s f = 0.52). There were no other significant main effects or interactions for the coherence conditions of 3%, 12%, 25% or 50%. Post-hoc tests were conducted to compare performance in the 6% coherence condition at each noise level against the baseline. All p-values were corrected for multiple comparisons. These comparisons revealed that a tRNS intensity of .25mA significantly enhanced motion discrimination performance relative to baseline (t(13) = 3.39 p_corrected_ < 0.02). A similar enhancement was evident for the 6% coherence level at an intensity of .375mA, but this effect did not survive our stringent correction for multiple comparisons, (t(13) = 2.53, p_corrected_ > 0.1). These results suggest that perceptual decision-making for sensory stimuli that are just below threshold can be improved by adding a small amount of neural noise over bilateral visual cortex, consistent with predictions arising from the stochastic resonance hypothesis [8].

We used the hierarchical drift diffusion model ((HDDM, [37]) to determine which aspect of decision-making was affected by tRNS. As shown in the right panel of Fig 3, the drift rate was markedly affected by tRNS for the 6% coherence condition, whereas it was relatively unaffected for the remaining coherence levels. We submitted the drift-rate parameter to a 5 x 4 repeated measures ANOVA. This analysis revealed a significant main effect of tRNS-intensity (F(3,39) = 2.85, p = 0.049) and of coherence level (F(4,52) = 3.18, p = 0.02 on drift rate, as well as a significant tRNS-intensity x coherence level interaction (F(12,156) = 3.22, p < .01, Cohen’s f = 0.47). To isolate the source of the significant interaction, one-way ANOVAs were conducted for each coherence level separately. Consistent with the behavioral data, there was a significant effect of tRNS intensity on the drift rate in the 6% coherence condition (F(3,39) = 5.63, p < .01, Cohen’s f = .58), but no significant effects for the other coherence levels were observed (3%, 12%, 25%, 50%). Adding higher amounts of noise to the 6% coherence condition resulted in a decrease in both behavioural performance and the drift rate (see Fig 3). This inverted U-shaped relationship between performance and noise level is a key signature of the stochastic resonance effect [8, 38].

Post-hoc tests were conducted to compare performance in the 6% coherence condition against the baseline for each noise level. For the tRNS intensity of .25mA, the drift rate for the 6% coherence condition was significantly higher than baseline (t(13) = 3.44, p_corrected_ < 0.02, corrected for multiple-comparisons). A similar benefit for the 6% coherence condition was apparent for the tRNS intensity of .375mA, but this effect did not survive correction for multiple comparisons (t(13) = 2.55, p = 0.1). Separate 5 x 4 repeated measures ANOVAs revealed no significant effects for the bound-separation parameter (all p > 0.06), and no significant effects for non-decision time (all p > 0.13).

**Fig 4:**
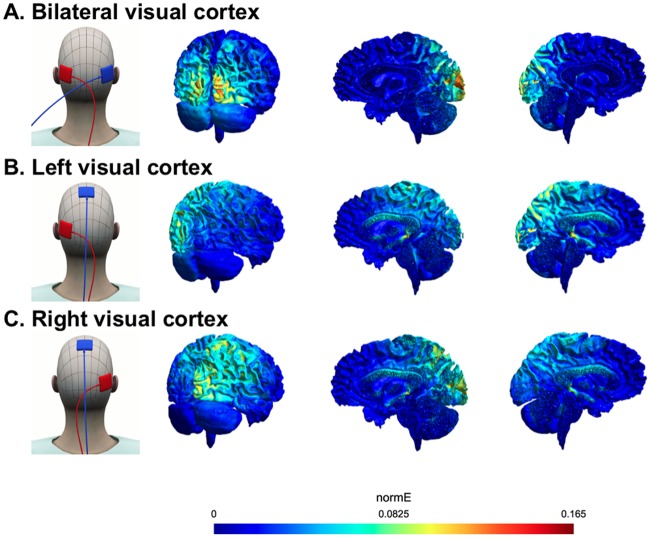
Electrode pad montages and modelled electrical field strength (normE) for each of the three tRNS experiments. **A**. Bilateral visual cortex stimulation (Experiment 1). **B**. Left visual cortex stimulation (Experiment 2). **C**. Right visual cortex stimulation (Experiment 3).

Previous studies of visual motion discrimination have shown reliable effects of offline transcranial electrical stimulation—as opposed to the online effects reported here—following unilateral stimulation of left or right visual cortex in isolation [34, 39, 40]. We therefore conducted two further experiments to determine whether the stochastic resonance effects we observed for bilateral tRNS in Experiment 1 also arise for unilateral visual stimulation. We also modelled the current spread for the electrode montage used in each experiment using the SimNibs toolbox [41]. The modelling results revealed that the bilateral electrode montage affected the visual cortex in both hemispheres, whereas the unilateral configurations affected one hemisphere (left or right) only (see Fig 4).

### Experiments 2 and 3—Effect of unilateral visual cortex stimulation on perceptual decision-making

**Fig 5:**
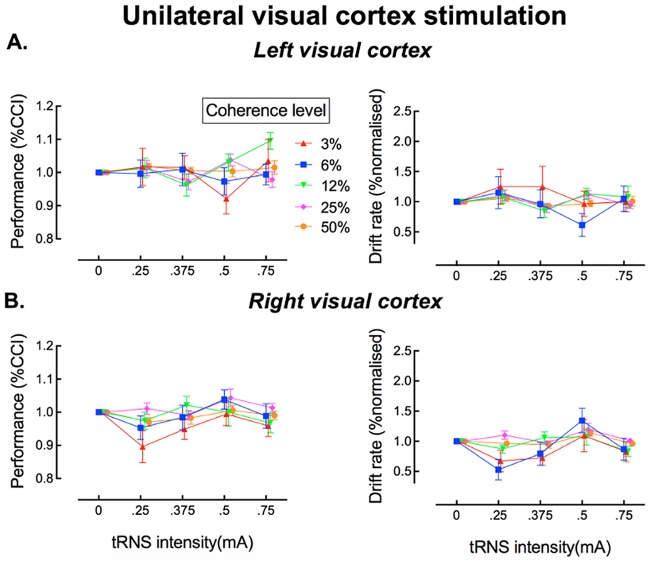
Effects of transcranial random noise stimulation (tRNS) on perceptual decision-making in the dot-motion discrimination task for unilateral stimulation of the left visual cortex (A) and right visual cortex (B). The left panels show performance for each motion coherence level as a function of tRNS intensity. The right panels show the drift rate derived from modelling of the data shown in the corresponding plots to the left.

Fig 5A and 5B show the behavioral results for Experiments 2 and 3. Neither left nor right unilateral tRNS influenced visual discrimination performance or the drift rate derived from the HDDM.

To characterize these data statistically, we employed the same analytic approach as in Experiment 1 (bilateral stimulation), for both the behavioral data and the drift diffusion modelling. There was no significant interaction between stimulation intensity and coherence level for either left unilateral or right unilateral visual cortex stimulation (p > .05 for all key comparisons). Thus, there was no evidence for a stochastic resonance effect as observed during bilateral stimulation in Experiment 1 (see also S1 Fig).

## Discussion

We found that adding an optimal amount of noise bilaterally to the visual cortex can enhance perceptual decision-making in a motion discrimination task, particularly for stimuli that are just subthreshold (6% coherence), as predicted by the stochastic resonance hypothesis [8]. By contrast, there was no reliable effect of tRNS on stimuli that were above or well below threshold, again consistent with the stochastic resonance account. When modeled as a drift-diffusion process, the tRNS-induced performance improvement for 6% coherence displays coincided with an increase in the rate of evidence accumulation for these displays only, reflected as a change in the model’s drift-rate parameter. The same model revealed no change in either bound-separation or non-decision time, suggesting that an optimal level of neural noise exclusively improves perceptual decision-making by enhancing sensory information quality, consistent with a stochastic resonance account [7–9]. Our results cannot be attributed to a speed-accuracy trade-off in observers’ responses, as the DDM controls for any such effects [42].

All tRNS intensities and motion coherence levels were randomized over participants to account for any fatigue, aftereffects or learning effects across conditions. It has been demonstrated that continuous application of at least 5-minutes of tRNS over the motor cortex can increase motor cortex excitability [43]. The effects we report here are unlikely to be due to changes in general cortical excitability, however, as it has previously been demonstrated that cathodal tDCS influences neuronal processing in motion sensitive areas, irrespective of the coherence level of visual stimuli [44]. By contrast, here we found a specific effect of tRNS on perceptual judgements for *subthreshold* motion coherence levels only.

There was no evidence for a stochastic resonance effect when noise was applied unilaterally to the visual cortex. The absence of an enhancement effect for unilateral tRNS was not due to differences in baseline performance between the groups: discrimination performance in the 6% coherence condition was similar across experiments (Experiment 1–60%; Experiment 2–58%; Experiment 3–57%). Modelling of the electrical field for each electrode montage (Fig 4) indicated a higher peak current when the tRNS was applied bilaterally than in the unilateral stimulation conditions. It is unlikely that this apparent difference in current densities prevented a stochastic resonance effect for the unilateral stimulation conditions, however, because the same absolute current densities during bilateral stimulation were also reached during unilateral stimulation, but simply at higher tRNS intensities (see S1 Table).

The visual stimuli employed in our motion discrimination task were always presented in the centre of the screen (i.e., at the fovea), and thus would have been processed initially by visual areas in both the left and right hemispheres [45] as early cortical areas receive visual information from the contralateral hemifield. It is also known that area V5/MT receives information from both visual hemifields [46, 47]. It is likely, therefore, that in the motion discrimination task employed here, areas V1 and V5/MT in both hemispheres would need to be recruited for successful performance. Based on our findings, it seems reasonable to hypothesise that visual areas in *both* hemispheres must be stimulated concurrently with tRNS for the stochastic resonance effect to occur. A study by Boulinguez and colleagues suggests that most human observers have a right hemisphere dominance for processing of visual motion stimuli, and non-invasive brain stimulation can enhance these individual asymmetries [48]. We did not test our participants for the presence of such asymmetries for visual motion perception, but it is possible that the absence of a stochastic resonance effect with unilateral stimulation was due to a mixture of right- and left-hemisphere dominant individuals in our sample.

Because of the relatively diffuse nature of transcranial electrical stimulation in general [49], it is not possible to determine the specific anatomical regions that mediate the stochastic resonance effect we observed. The primary visual cortex (V1) [50] and motion area V5/MT are both crucial for the processing of dynamically moving visual stimuli [51–53]. These two areas are highly interconnected, so our bilateral stimulation protocol might have impacted motion processing in area V5/MT, enhanced signal quality in area V1, or both. Further work using more focal stimulation techniques (e.g., transcranial magnetic stimulation) will be needed to pinpoint the visual areas responsible for the stochastic resonance effects we report here.

Recently, animal work has shown that optogenetic-noise-photostimulation of the barrel cortex in mice enhances both evoked-field and spike-firing responses to mechanical stimulation of the whiskers [54, 55]. Optogenetic-noise-photostimulation could be used in combination with a decision task in mice [56] to further investigate the mechanism underlying our observed behavioral effect in human perceptual-decision making.

Our results are in line with recent work which employed a similar motion-discrimination task to show that decision-making is sensitive to the addition of external noise to visual motion stimuli [28]. Future studies could investigate whether there is an interaction between external noise added to a visual motion stimulus, as used in [28], and central noise delivered via tRNS over the visual cortex. If external and central noise affect a common underlying mechanism, then their combination should yield an interacting influence on the SR effect. By contrast, if external and central noise have separate underlying causes, their influence on the SR effect should vary independently.

Our findings suggest that a stochastic resonance effect can be induced in a decision-making task when noise is directly applied to the visual cortex with tRNS [24, 25]. Moreover, ours is the first study to show that this stochastic resonance effect enhances the quality of information processing as indicated by an accelerated rate of evidence accumulation. Many daily activities depend on our ability to decide upon appropriate actions based on available sensory information, e.g., judging the speed of oncoming traffic to decide whether it is safe to cross the road. Even subtle impairments of perceptual decision making are likely to have a negative impact on daily functioning. Our findings could be applied to enhance perceptual decision making in people with developmental [57] or acquired [58] neurological impairments, in the elderly [59], or even potentially amongst those in specialised professional and sports settings.

## Materials and methods

### Ethics

The study was approved by The University of Queensland Human Research Ethics Committee and the Kantonale Ethik Komission Zurich, Switzerland, and was conducted in accordance with the Declaration of Helsinki.

### Participants

To select an appropriate sample size for the study, we conducted a power analysis (G*Power version 3.1.3, [60]). This indicated that a sample of 10 participants would be sufficient to detect a significant effect on discrimination performance in a repeated-measures ANOVA with a power of 0.80 for an α level of 0.05. This estimate was based on an effect size (Cohen’s *d*: 0.77) derived from our previous work on the influence of tRNS on detecting weak visual signals [18]. We chose to err on the side of caution, and tested 15 participants in each of the three experiments (bilateral, unilateral left and unilateral right stimulation). Thus, a total of 45 healthy adults participated in the study overall (28 males, aged: 18–27 years, mean age = 22.5 years). All participants had normal or corrected-to-normal vision, and met the inclusion criteria for tRNS as assessed by a checklist prior to the experiment [61]. Written informed consent was obtained for all participants.

### Transcranial random noise stimulation (tRNS)

Each participant received four tRNS noise intensities twice (.25mA, .375mA, .5mA and .75mA; all delivered at frequencies between 100 and 640 Hz). Noise intensity order was randomized across participants. The tRNS was applied with a 0mA offset, and was applied for 20 trials followed by 20 trials of no-stimulation. This order was counterbalanced across participants. The tRNS was delivered via a battery-driven electrical stimulator (version DC-Stimulator PLUS, neuro-Conn). The maximum current density was 46.87 μA/cm2, which is well within published safety limits [60]. Electroconductive gel was applied to the contact side of the electrode (4 x 4 cm) to reduce skin impedance. In Experiment 1, the visual cortex was stimulated bilaterally, with electrodes placed 3.5 cm above the inion and 6.5 cm left and right of the midline in the sagittal plane. These coordinates were selected based on previous brain imaging and stimulation studies that investigated the offline aftereffects of transcranial current stimulation (tCS) on a motion detection task [35, 62–66]. In Experiment 2, the stimulation electrode was placed over the left visual cortex (positioned as described for Experiment 1), and the reference electrode was placed over the vertex (Cz in the 10–20 EEG-system). In Experiment 3, the stimulation electrode was placed at the homologous location over the right visual cortex, and the reference electrode was placed at the vertex as in Experiment 2.

### Visual decision-making task

All experiments took place in a dark and quiet room. Visual stimuli were generated using Matlab 8.0 (2012b) and the Psychophysics Toolbox [67–69], and were presented using a Dell PC (T3400) running Windows XP with a NVIDIA Quadro FX 1700 graphics card. Stimuli were presented on an Asus VG428QE color monitor with a resolution of 1920x1080 pixels, and a refresh rate of 60 Hz. The luminance of the monitor was gamma-corrected with a maximum intensity of 316.5 cd/m^2^ and minimum of 0.33 cd/m^2^. Viewing distance was maintained at 62 cm using a chinrest, meaning the display subtended 46° x 27° (1.5’ per pixel). We employed a two-alternative, forced-choice random-dot motion discrimination task [51, 70] in which participants judged the direction (leftward or rightward) of the coherently moving dots as quickly and as accurately as possible. Each block of 20 trials began with the presentation of a central fixation cross (2 s). On each trial, the fixation cross was presented for 200 ms. The motion stimulus then appeared, and consisted of 100 square dots (3 x 3’) within an aperture (radius 4.1°) at the centre of the screen. The dots were randomly positioned within the aperture on the first frame before rigidly translating at 1.5 deg/s. If a dot was going to move outside the aperture on the next frame, it was wrapped to the opposite side of the aperture. The dot-motion display remained visible until response, up to a maximum duration of 3 s. Participants indicated their choices by pressing the left or right ‘shift’ key on a standard keyboard with the left or right index finger, respectively. If the participants did not respond within 3 s, the motion stimulus was extinguished, and the trial was counted as incorrect and excluded from further analysis. Participants were provided with immediate auditory feedback. A low-pitched tone indicated a correct response, a high-pitched tone an incorrect response, and a prolonged low-pitched tone indicated a response that was too slow (i.e., > 3 s). A new trial commenced 2 s after the previous response.

A method of constant stimuli was used to determine global motion sensitivity. A proportion of the dots moved coherently to the left or right, and the remaining dots moved in random directions. Thus, for example, a coherence level of 3% indicates a display in which 3% of the dots translated coherently (left or right, depending on the trial), while the remaining 97% of dots moved in random directions. Five logarithmically spaced coherence levels (3%, 6%, 12%, 25% and 50%) were chosen, consistent with previous work [71]. The dots had a limited lifetime of 5 frames. In keeping with a common convention [72], half of the dots were black and half of the dots were white, all of which were presented on a mid-gray background.

To measure the effects of tRNS on visual motion discrimination, participants performed 10 blocks of 200 trials each, with different tRNS intensities. The first and the last blocks contained no tRNS. The four tRNS levels (.25mA, .375mA, .5mA and .75mA) were applied twice each in blocks 2–9, in random order. The first block served as practice, and the data obtained were not included in the analyses. Each block contained 200 motion discrimination trials, with an equal number of presentations of each motion coherence level, presented in a pseudo-randomized order (the total length of each block was ~ 6 mins). To minimize any build-up of tRNS effects, stimulation was applied for 20 trials before being turned off for the next 20 trials within each block. Coherence levels for stimulator-on and stimulator-off trials were balanced for each observer, and were combined during data analysis. Including electrode setup and data collection, the entire experiment took around 90 minutes per participant.

### Data analysis

The same statistical procedures were used in all three experiments. In each experiment, one participant was excluded (3 in total) because the individual did not reach 80% correct responses in the highest coherence condition. The α level was set to 0.05 for all tests, adjusting for multiple comparisons using the Bonferroni correction. We used the same procedure to quantify the stochastic resonance effect as in our previous paper [18]. By normalizing the data to the mean of the noise-free trials, which were interspersed with active tRNS trials throughout the experiment, we were able to rule out any contribution from practice, learning or fatigue. The normalized behavioral data were subjected to a repeated-measures ANOVA with the factors of coherence level (5 levels: 3%, 6%, 12%, 25% and 50%) and tRNS-intensity (4 levels: .25mA, .375mA, .5mA and .75mA).

### Computational modelling

Drift diffusion modeling (DDM) has been employed widely to disentangle the different component processes involved in simple decision-making tasks [29, 73]. It captures three distinct stages of the decision process: (i) boundary separation, which indicates how much evidence must be accumulated before a response is made; (ii) information accumulation rate (‘drift rate’), which is a measure of how rapidly evidence is accumulated and depends on the quality of evidence in the stimulus, such that easier decisions result in a higher drift rate; and (iii) non-decision time, which is the time required to encode the stimulus and execute an appropriate motor response [29]. We used the hierarchical drift diffusion model (HDDM) to fit the DDM parameters to the data [37]. The HDDM uses a Bayesian method for estimating the DDM parameters, which allows simultaneous estimation of group and subject parameters. A benefit of the HDDM is that it outperforms other approaches when a small number of trials is available [74].

We took a similar approach in our implementation of the HDDM as Herz and colleagues [75]. We fixed the starting parameter, *z* (also known as the bias parameter), to 0.5, which is chance level in a 2-AFC task. We modelled the data with the drift rate, bound separation and non-decision time as free parameters. We obtained parameter estimates for the conditions noise-on/noise-off, coherence level and tRNS intensity. We normalized the obtained parameters to the zero-noise (no tRNS) trials. This normalization procedure was the same as for the correct choice index (CCI) data. Markov-chain Monte Carlo sampling methods were used for accurate Bayesian approximation of the posterior distribution of parameters (generating 20,000 samples, discarding 10,000 samples as burn-in, and keeping every fifth subsequent sample). We visually inspected all traces of model parameters, their autocorrelation and computed the R-hat (Gelman-Rubin) convergence statistics to ensure that the models had properly converged [37]. All R-hat values were below 1.1, verifying that convergence had been achieved [76]. For each experiment, we plotted observed and predicted RTs for the 10, 30, 50, 70 and 90 percentile of trials (i.e., for the fastest 10% of trials, fastest 30% of trials, etc.) against the cumulative probability (see S2 Fig). These results indicated that the HDDM provided a good prediction of the observed data. The parameter estimates for bound separation and non-decision time (NDT) are shown in S1 Fig. As a sanity check we also plotted the drift rate against motion coherence level (S3 Fig). As expected, the drift rate increased with increasing motion coherence. This provides further confirmation that our model provided an appropriate fit to the data.

### Current modelling

We used the SimNibs toolbox to model current flow in the brain [41]. The modelling results revealed that the bilateral electrode setup affected the visual cortex in both hemispheres, whereas the unilateral stimulation affected one hemisphere (left or right) only (see Fig 4). The SimNibs modelling approach does not provide any frequency-specific information. To determine whether the chosen tRNS frequencies (100–640 Hz) reached the cortex, we estimated the electrical field strength at frequencies between 100 and 500 Hz, in steps of 50 Hz, with Spheres 2.0 [77]. The estimated electrical field strengths can polarize somatic membranes (polarization <0.3 mV per V/m electrical field [78]) and modulate network activity at low stimulation intensities (0.2 V/m, [79, 80]). The electrical field strengths obtained with this modelling approach are estimates of the amount of current that reached the cortex (see S1 Table). A recent study suggested that these results might be overestimated due to possible inaccurate resistance estimates for different tissues [81], but even very low electrical fields (0.2 V/m) are able to influence network activity.

### Additional analysis

Analysis of the baseline data in all three experiments revealed no significant interaction between coherence level and tRNS intensity (repeated-measures ANOVA with a within-subjects factor of coherence level and between-subjects factor of experiment, F(2,39) = 1.15, p > .32), suggesting that the stochastic resonance-effect observed in Experiment 1 was not driven by differences in baseline performance between the three experiments. Across all three experiments, there was a highly significant main effect of coherence level on performance, as expected. For completeness, we also report here a small number of significant main effects which seem to be unrelated to the central stochastic resonance hypothesis under examination in this study. First, there was a small but consistent main effect of tRNS intensity on accuracy during right visual cortex stimulation, F(3,39) = 3.13 p = .036, Cohen’s f = 0.49. Post-hoc contrasts revealed that this effect was driven by overall poorer performance for the .25mA tRNS intensity, regardless of motion coherence level, t(69) = -2.78 p_corrected_ < 0.03. This decrease in performance was mirrored by a significant main effect of tRNS-intensity on drift rate (see S1 Fig), (F(3,39) = 4.54 p < .01, Cohen’s f = 0.59, which was again specific to the .25mA tRNS intensity, (t(69) = 2.67 p_corrected_ = .04), regardless of motion coherence level. Second, there was a significant main effect of coherence level on bound-separation during stimulation of the right visual cortex, F(4,52) = 3.09 p = .024, Cohen’s f = 0.4 (see S1 Fig). Post-hoc tests showed that the bounds were significantly closer together for the highest (50%) coherence condition, t(55) = 3.16 p_corrected_ < .04, relative to baseline), but there were no significant effects on bound separation for the other coherence levels. Although these effects were statistically significant, there was no interaction between tRNS-intensity and stimulus coherence level, which is a hallmark of the stochastic resonance effect. Moreover, it is important to note that these unspecific effects only occurred for right visual cortex stimulation. In that experiment there was no evidence for a stochastic resonance effect.

## Supporting information

S1 Fig The HDDM results for the bounds and non-decision times (NDT) for Experiments 1–3. **(A)** Bilateral visual cortex stimulation. **(B)** Left visual cortex stimulation. **(C)** Right visual cortex stimulation. In the right unilateral stimulation condition, there was a significant main effect of coherence level on bound-separation F(4,52) = 3.088, p = 0.024, Cohen’s f: 0.4. Post-hoc tests showed that the bounds were significantly closer together for the highest (50%) coherence condition, t(55) = -3.157, p < .01. There were no other significant effects for the bounds or non-decision times. *p_corrected_ < 0.05.
(TIFF)

S2 Fig Quantile probability plots of mean response times in the motion discrimination task, plotted separately for the three stimulation conditions in Experiments 1, 2 and 3. **(A)** Bilateral visual cortex stimulation. (B) Left visual cortex stimulation. **(C)** Right visual cortex stimulation. Observed response times for five quantiles (10, 30, 50, 70 and 90%) are shown in blue, plotted as a function of their cumulative probability. Red symbols show predicted quantile means, with error bars indicating the standard deviation of the posterior predictive distribution of the model. The plots show that the hierarchical drift diffusion model provides a good fit to the data, and that the mean response times are comparable across the experiments.
(TIFF)

S3 Fig Plot of average drift rates in the bilateral visual cortex stimulation condition for different motion-coherence levels. As expected the drift rate increases with increasing coherence.
(TIFF)

S1 Table Modelled electrical field strengths for different transcranial electrical current stimulation intensities and frequencies. The modelling suggests that all stimulation frequencies were transmitted to the brain, and that the current applied was of sufficient intensity to reach the cortex (V/m). Neuronal membranes can be polarized by 0.3 mV per V/m electrical field strength [78] and network activity can be modulated at intensities from 0.2 V/m [79, 80]).
(DOCX)
