# Supplementary figures and images for: Stochastic resonance enhances the rate of evidence accumulation during combined brain stimulation and perceptual decision-making
Source: PLoS Comput Biol. 2018 Jul 18;14(7):e1006301. doi: 10.1371/journal.pcbi.1006301 (PMC6066257; doi:10.1371/journal.pcbi.1006301)

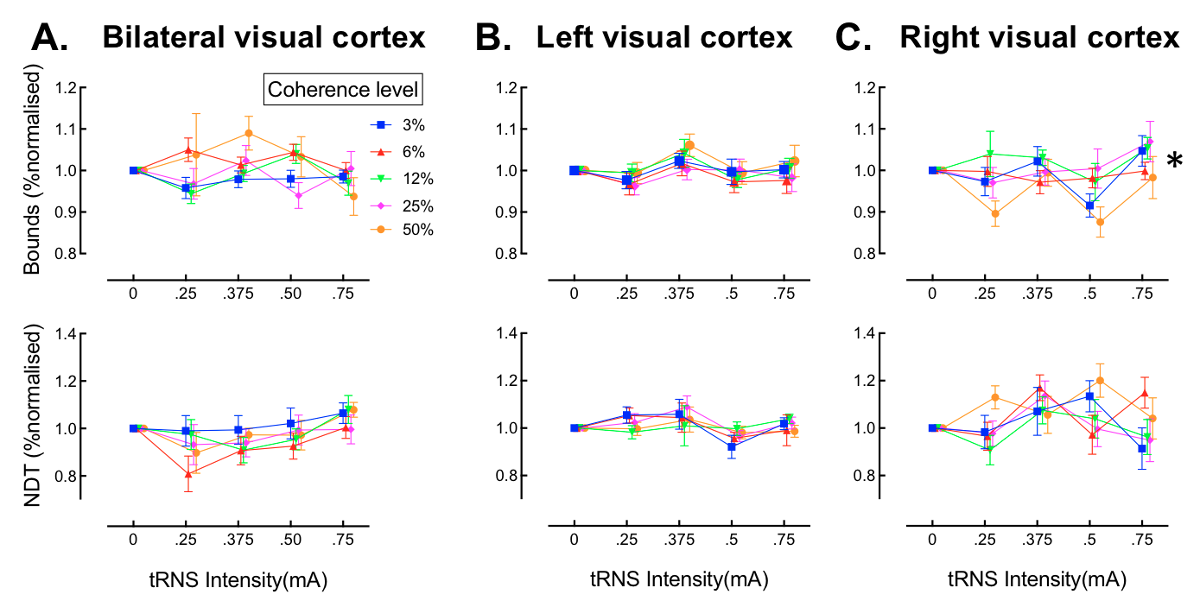

Supplement: S1 Fig — (A) Bilateral visual cortex stimulation. (B) Left visual cortex stimulation. (C) Right visual cortex stimulation. In the right unilateral stimulation condition, there was a significant main effect of coherence level on bound-separation F(4,52) = 3.088, p = 0.024, Cohen’s f: 0.4. Post-hoc tests showed that the bounds were significantly closer together for the highest (50%) coherence condition, t(55) = -3.157, p < .01. There were no other significant effects for the bounds or non-decision times. *pcorrected < 0.05. (TIFF) [file pcbi.1006301.s001.tiff]

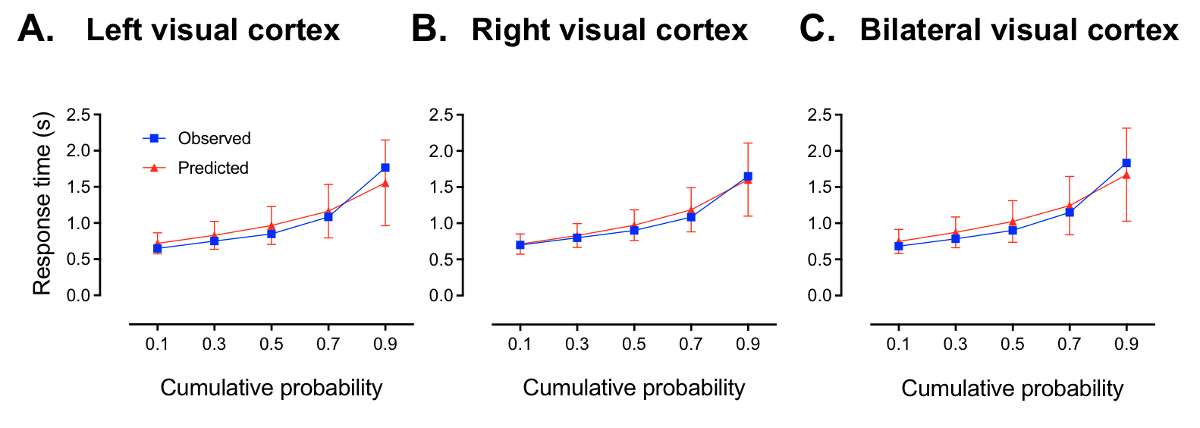

Supplement: S2 Fig — (A) Bilateral visual cortex stimulation. (B) Left visual cortex stimulation. (C) Right visual cortex stimulation. Observed response times for five quantiles (10, 30, 50, 70 and 90%) are shown in blue, plotted as a function of their cumulative probability. Red symbols show predicted quantile means, with error bars indicating the standard deviation of the posterior predictive distribution of the model. The plots show that the hierarchical drift diffusion model provides a good fit to the data, and that the mean response times are comparable across the experiments. (TIFF) [file pcbi.1006301.s002.tiff]

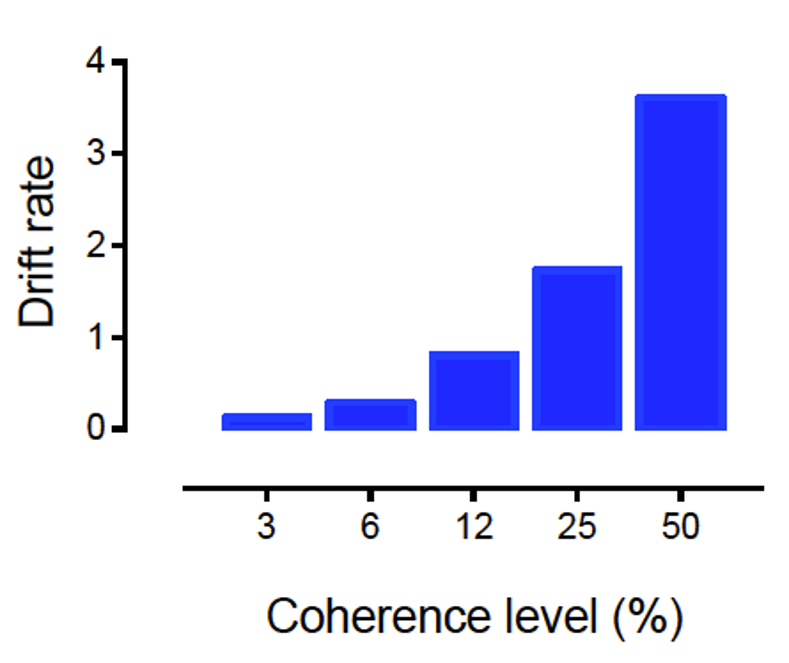

Supplement: S3 Fig — As expected the drift rate increases with increasing coherence. (TIFF) [file pcbi.1006301.s003.tiff]
