# Supplementary material for: Stochastic resonance enhances the rate of evidence accumulation during combined brain stimulation and perceptual decision-making
Source: PLoS Comput Biol. 2018 Jul 18;14(7):e1006301. doi: 10.1371/journal.pcbi.1006301 (PMC6066257; doi:10.1371/journal.pcbi.1006301)
Supplement: S1 Table — The modelling suggests that all stimulation frequencies were transmitted to the brain, and that the current applied was of sufficient intensity to reach the cortex (V/m). Neuronal membranes can be polarized by 0.3 mV per V/m electrical field strength [78] and network activity can be modulated at intensities from 0.2 V/m [79, 80]). (DOCX) [file pcbi.1006301.s004.docx]

**Supporting information**

**Supplemental Table**

|  | **Bilateral stimulation** | | | | **Unilateral stimulation** | | | |
| --- | --- | --- | --- | --- | --- | --- | --- | --- |
| **Frequency (Hz)** | *.25mA*  *(V/m)* | *.375mA*  *(V/m)* | *.5mA*  *(V/m)* | *.75mA*  *(V/m)* | *.25mA*  *(V/m)* | *.375mA*  *(V/m)* | *.5mA*  *(V/m)* | *.75mA*  *(V/m)* |
| *100* | .85 | 1.25 | 1.63 | 2.6 | .85 | 1.25 | 1.63 | 2.6 |
| *150* | .85 | 1.25 | 1.63 | 2.5 | .85 | 1.25 | 1.63 | 2.5 |
| *200* | .85 | 1.25 | 1.62 | 2.5 | .84 | 1.25 | 1.62 | 2.5 |
| *250* | .84 | 1.24 | 1.62 | 2.5 | .84 | 1.24 | 1.62 | 2.5 |
| *300* | .84 | 1.24 | 1.62 | 2.5 | .84 | 1.24 | 1.62 | 2.5 |
| *350* | .84 | 1.24 | 1.62 | 2.5 | .84 | 1.24 | 1.62 | 2.5 |
| *400* | .84 | 1.23 | 1.62 | 2.5 | .83 | 1.23 | 1.62 | 2.5 |
| *450* | .84 | 1.23 | 1.62 | 2.5 | .83 | 1.23 | 1.62 | 2.5 |
| *500* | .84 | 1.23 | 1.62 | 2.5 | .83 | 1.23 | 1.62 | 2.5 |
